# A Novel Necroptosis-Related Prognostic Signature of Glioblastoma Based on Transcriptomics Analysis and Single Cell Sequencing Analysis

**DOI:** 10.3390/brainsci12080988

**Published:** 2022-07-26

**Authors:** Yiwen Wu, Yi Huang, Chenhui Zhou, Haifeng Wang, Zhepei Wang, Jiawei Wu, Sheng Nie, Xinpeng Deng, Jie Sun, Xiang Gao

**Affiliations:** 1 Department of Neurosurgery, Ningbo First Hospital, Zhejiang University School of Medicine, Ningbo 315010, China; wuyiwen@zju.edu.cn (Y.W.); zhouchenhui1989@126.com (C.Z.); 18857496593@139.com (H.W.); fagoce@126.com (Z.W.); 21818268@zju.edu.cn (J.W.); niesheng@163.com (S.N.); 22118158@zju.edu.cn (X.D.); nbyysj@sina.com (J.S.); 2 Key Laboratory of Precision Medicine for Atherosclerotic Diseases of Zhejiang Province, Ningbo 315010, China; 3 Medical Research Center, Ningbo First Hospital, Ningbo 315010, China

**Keywords:** glioblastoma, necroptosis, machine learning algorithms, single-cell analysis, immune microenvironments

## Abstract

Background: Glioblastoma (GBM) is the most common and deadly brain tumor. The clinical significance of necroptosis (NCPS) genes in GBM is unclear. The goal of this study is to reveal the potential prognostic NCPS genes associated with GBM, elucidate their functions, and establish an effective prognostic model for GBM patients. Methods: Firstly, the NCPS genes in GBM were identified by single-cell analysis of the GSE182109 dataset in the GEO database and weighted co-expression network analysis (WGCNA) of The Cancer Genome Atlas (TCGA) data. Three machine learning algorithms (Lasso, SVM-RFE, Boruta) combined with COX regression were used to build prognostic models. The subsequent analysis included survival, immune microenvironments, and mutations. Finally, the clinical significance of NCPS in GBM was explored by constructing nomograms. Results: We constructed a GBM prognostic model composed of NCPS-related genes, including CTSD, AP1S1, YWHAG, and IER3, which were validated to have good performance. According to the above prognostic model, GBM patients in the TCGA and CGGA groups could be divided into two groups according to NCPS, with significant differences in survival analysis between the two groups and a markedly worse prognostic status in the high NCPS group (*p* < 0.001). In addition, the high NCPS group had higher levels of immune checkpoint-related gene expression, suggesting that they may be more likely to benefit from immunotherapy. Conclusions: Four genes (CTSD, AP1S1, YWHAG, and IER3) were screened through three machine learning algorithms to construct a prognostic model for GBM. These key and novel diagnostic markers may become new targets for diagnosing and treating patients with GBM.

## 1. Introduction

Glioblastoma (GBM) is an aggressive, highly malignant, and lethal brain tumor that originates from astrocytes and accounts for approximately 80% of all CNS malignancies [1]. The overall survival of GBM patients after standard treatment is generally less than 15 months, and although some patients with recurrent GBM can benefit from improved survival and prognosis when they undergo secondary surgery [2,3], the overall 5-year survival rate is still less than 5% [4,5]. The high frequency of molecular mutations (p53, RB, unmethylated MGMT) makes GBM less sensitive to conventional cytotoxic therapies and highly resistant to standard chemotherapy and radiotherapy [6]. Thus, it is important to identify new targets and molecular mechanisms that can propose alternative therapeutic strategies. Genetic data mining has attracted more and more attention with the development of high-throughput sequencing and bioinformatics. Therefore, the discovery of new markers that inhibit cell growth, migration, and invasion in GBM, and the study of their biological mechanisms for treating GBM patients, as well as the construction of prognostic models are undoubtedly essential for the advancement of prognosis and treatment.

Tumors can escape from immune surveillance, which includes the loss of programmed apoptosis [7]. Therefore, the inducing of programmed apoptosis in tumor cells is an important treatment for tumors. Targeting NCPS is considered a prospective cancer treatment strategy. Programmed cell death participated in homeostatic regulation, inflammatory response, anti-infective, and carcinogenic, which includes apoptosis, cell scorch, NCPS, and iron death [8,9,10]. Since the discovery of NCPS by HITOMI et al. in 2008 [11], the underlying mechanisms have been elucidated as research progresses [12]. Further exploration of NCPS has contributed to a new understanding of the composition of programmed cell death. It is mechanistically similar to apoptosis and morphologically similar to necrosis. It is mediated by activated receptor-interacting protein kinase 1 (RIPK1), RIPK3, and mixed-spectrum kinase domain-like (MLKL) [13]. Previous studies find NCPS associated with several tumor types and their immune microenvironment [14]. At present, research on NCPS in neurological disorders has focused on non-neoplastic diseases, such as cerebral ischemia [15], traumatic brain injury [16], and Alzheimer’s disease [17]. Previous researchers have shown that Alzheimer’s disease can be alleviated by overexpression of the core molecule of NCPS, MLKL [17]. Damage caused by cerebral ischemia can be reduced by inhibitors of NCPS [15]. However, its relationship with GBM is not yet clear. Thus, it is of great significance to explore the role of NCPS in GBM.

Single-cell RNA sequencing (scRNA-seq) is now emerging as a new approach to the study of human tumors, allowing gene expression data to be analyzed at the individual cell level. The multidimensional analysis of cell clustering allows for a clear exploration of the cellular heterogeneity of tumor tissue and the spatial, molecular, and functional heterogeneity of associated immune cells [18]. Currently, some researchers have begun to explore glioma heterogeneity from a single-cell perspective [19,20,21]. However, the majority of studies have focused on the heterogeneity of immune cells themselves in gliomas, with limited studies focusing on the expression of cell death-related molecules in gliomas and the impact on the immune microenvironment, particularly NCPS.

Nowadays, next-generation sequencing (NGS) technologies allow us to explore the variation of thousands of molecules at multiple omics levels, but how to choose the right and accurate variables in a high-dimensional computation, machine learning is a promising algorithm for computing such problems [22]. Some of the popular machine learning algorithms include the Least Absolute Shrinkage and Selection Operator (LASSO), Support Vector Machine (SVM), and Random Forest (RF). LASSO [23] is a regression method that is of great importance in datasets with many variables and supports unbiased parameter selection. SVM is an algorithm that efficiently handles problems by using a non-linear kernel function [24,25], while recursive feature elimination (RFE) is a popular method of variable selection at the multi-omics level [26,27]. In this research, we used support vector machine recursive feature elimination (SVM-RFE) to dimensionally reduce the search for suitable variables. Random Forest is an integrated algorithm based on decision trees [28]. The Boruta algorithm is a wrapper around the Random Forest algorithm, allowing the algorithm to iteratively remove less relevant features determined by statistical tests [29].

In this study, to explore GBM-associated potentially prognostic NCPS genes and clarify their functions to provide a new reference for the diagnosis and treatment of GBM patients, we collected and collated GBM patients’ data from The Cancer Genome Atlas (TCGA) database, the GEO database, and sequencing data from China Glioma Genome Atlas Database (CGGA). Weighted co-expression network analysis (WGCNA) combined with machine learning was used to develop a GBM NCPS-related prognostic model, which was divided into two groups based on median values according to risk scores. In addition, the value of using multiple immune scoring algorithms to assess the tumor immune microenvironment and tumor mutational load characteristics is also explored in this paper.

## 2. Materials and Methods

### 2.1. Transcriptome Data Download and Processing

The transcriptome files of GBM and the clinical information of the samples corresponding to them were downloaded from the TCGA database (https://portal.gdc.cancer.gov/, accessed on 16 May 2022), and the TPM data were extracted for subsequent analysis. After removing the non-primary tumor samples, a final sample of 143 GBM patients with complete clinical information was used as a training cohort (Table S1). After downloading 325 glioma samples from the CGGA portal (http://www.cgga.org.cn/index.jsp, accessed on 16 May 2022), only GBM-related information was retained, and, finally, 85 GBM samples were obtained, and the transcriptome FPKM data were converted to TPM data and combined with clinical information as the validation cohort data [30].

### 2.2. Single-Cell Data Download and Processing

The GSE182109 single-cell glioma dataset was downloaded from the GEO database (https://www.ncbi.nlm.nih.gov/geo/, accessed on 19 May 2022) and 11 GBM samples from 44 glioma patients were selected for analysis [31]. To assure data quality, the cells selected for this study set the screening criteria as less than 20% of mitochondrial genes, more than 200 genes, and gene expression between 200 and 7000, and containing more than three genes. The number of highly variant genes was 3000. Samples were integrated using SCT correction. Next, data dimensionality was reduced by setting the “DIMS” parameter to 25 and using the TSNE method. The clustering of cells was performed using the “KNN” method. Cells were subsequently annotated using different cell surface markers. Finally, NCPS genes were imported using the “AddModuleScore” function to determine the percentage of NCPS-related genes in each cell.

### 2.3. The Acquisition of NCPS-Related Genes

A total of 614 genes associated with NCPS were identified in the Genecards database (https://www.genecards.org/, accessed on 17 April 2022). We set the correlation coefficient threshold to greater than or equal to 1.0 and finally obtained 91 genes associated with NCPS.

### 2.4. Weighted Co-Expression Network Analysis (WGCNA) and Single Sample Gene Set Enrichment Analysis (ssGSEA)

Weighted gene co-expression network analysis (WGCNA) is a systems biology approach that explores the gene network itself and its associated phenotypes, as well as the core genes in the network, by finding highly correlated gene modules, which are ultimately used to identify candidate biomarker genes or therapeutic targets. Additionally, ssGSEA analysis is commonly used to quantify specific genomes in a sample. In this study, ssGSEA analysis was used to obtain the associated scores of NCPSfor each GBM patient. Then, WGCNA was used to obtain GBM score-associated gene modules in order to identify NCPS-associated genes.

### 2.5. Construction of the Prognostic Model Associated with NCPS

Univariate Cox analysis was used to initially identify the correlation between NCPS-related genes and patient prognosis, and we selected a *p* value less than 0.01 as an indication that the gene had a significant prognostic ability. After filtering, candidate prognostic genes were selected by a combined analysis of three machine learning algorithms, including the LASSO regression algorithm, the SVM-RFE algorithm [32], and the random forest-based Boruta algorithm [33]. In all three algorithms, the number of random seeds is set to 2022, the maximum number of passes of the lambda value on the data in LASSO is 1000, the 10-fold cross-validation is used in the RFE algorithm and the calculation method is set to “svmRadial”, and the number of trees to 500 is adjusted in the Boruta algorithm. Subsequently, overlapping genes from the three algorithms were selected from the candidate genes and, finally, a prognostic model was constructed using multivariate Cox regression. The correlation coefficients from multifactorial Cox regression analyses allowed us to derive the NCPS scores for each sample and to divide them into two groups according to the median. The prognosis was assessed using the Kaplan–Meier curve, and *p* < 0.05 was considered a clinically significant prognosis. The classification of GBM was evaluated by applying principal component analysis (PCA) to observe the grouping of this model. On this basis, modeling tests were performed on the external data using the above-mentioned methods.

### 2.6. Immunological Function and Mutation Analysis

The “IBOR” package integrates various immune infiltration assessment algorithms in the R language [34]. Using the “IBOR” package, we evaluated seven immune infiltration algorithms for GBM patients in the TCGA repository and showed the different levels of infiltration of various immune cells in a heat map to investigate the differences in immune cell infiltration between the different necroptotic groups. The genes associated with the immune checkpoint identified in the NCPS subgroup were also analyzed in a box plot. We also performed a study of the major variants in GBM, presenting the top 20 genes with the highest mutation frequency and the base types of the major mutations in GBM.

### 2.7. Location and Expression of Prognostic Models in Single-Cell Sequencing Analysis

In single-cell sequencing analysis, the expression of the genes used to construct the prognostic model was analyzed in the well-annotated cells to find out the expression of a specific gene in prognostic model genes at the single-cell level, and the expression was presented using TSNE plots.

### 2.8. Building a Predictive Nomogram

In this study, we used the Nomogram to establish a method that can effectively predict the probability of survival of patients, taking into account the influence of clinical covariates. The NCPS values were correlated with clinical data to construct a Nomogram to evaluate the risk of death in patients with GBM. Finally, the patient’s prognosis was evaluated based on the ROC curve.

## 3. Results

### 3.1. Single Cell Sequencing Data Analysis

**Figure 1:**
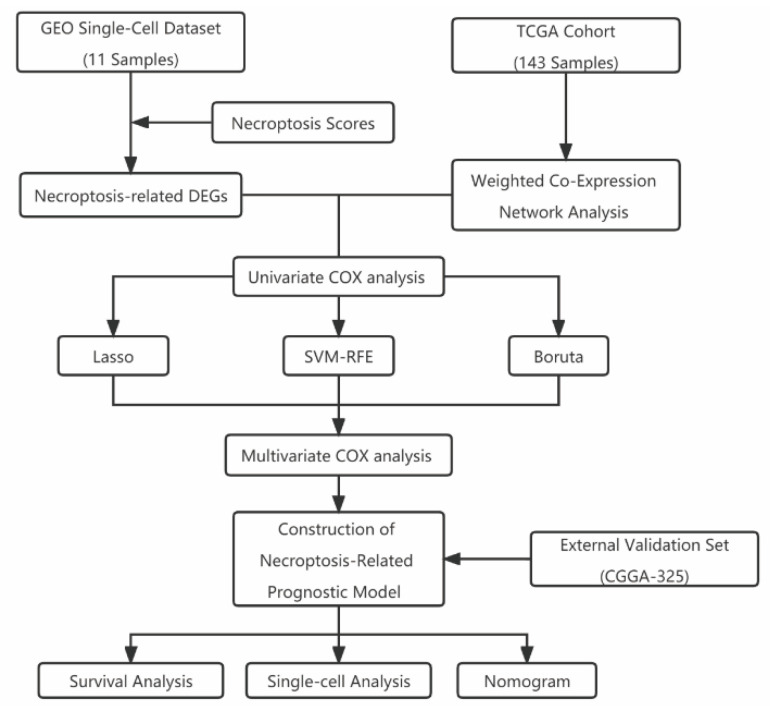
Flow chart.

**Figure 2:**
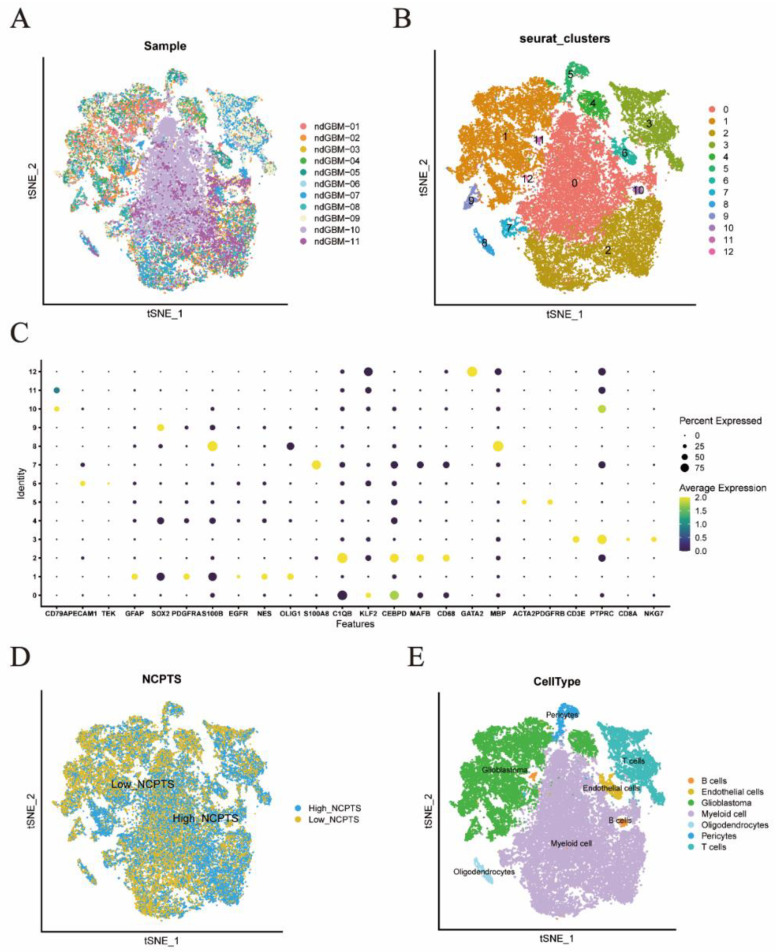
Single-cell sequencing analysis of GSE182109. (**A**) The integration effect of 11 samples is good. (**B**,**C**) Dimensionality reduction and cluster analysis. All cells in 11 samples were clustered into 12 clusters. (**D**) The percentage of necroptosis genes in each cell. The cells were divided into high- and low-necroptosis cells. (**E**) According to the surface marker genes of different cell types, the cells are annotated as B cells, T cells, endothelial cells, myeloid cells, pericytes, oligodendrocytes, and glioblastoma cells, respectively.

The study flow chart of this article is shown in Figure 1. The single-cell sequencing dataset GSE182109 from GBM was used as a follow-up analysis and different samples were integrated. The results are shown in Figure 2A, with 11 samples having no significant batch effects for subsequent analysis. All cells were then clustered into 12 clusters using the KNN clustering algorithm (Figure 2B,C). Subsequently, using the “AddModuleScore” function, 91 genes associated with NCPS were entered, resulting in the percentage of each cell type associated with NCPS. Based on the percentage of cells obtained, these cells were divided into two groups according to the median value and displayed as TSNE plots (Figure 2D). Then, based on the surface marker genes of different cell types (Table S2), their expression in different clusters was observed (Figure 2C), and, finally, seven cell types were identified based on the expression, they were: B cells, T cells, endothelial cells, myeloid cells, pericytes, oligodendrocytes, and glioblastoma cells (Figure 2E). The differentially expressed genes were then analyzed for the high and low NCPS groups, and a total of 2451 differential genes were identified.

### 3.2. Weighted Co-Expression Network Analysis

**Figure 3:**
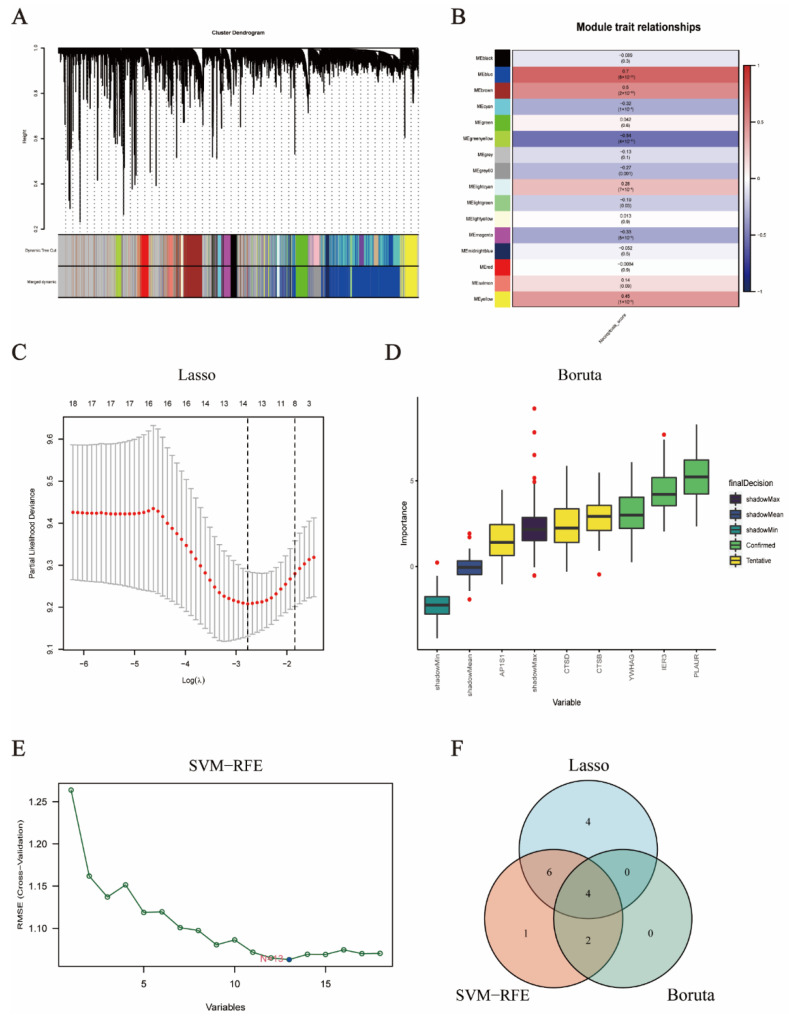
WGCNA and construction of the necroptosis-related prognostic model. (**A**,**B**) WGCNA found that Meblue, Mebrown, and Meyellow modules were closely related to the score of necroptosis. (**C**) Fourteen genes were selected to construct the prognostic model by Lasso regression. (**D**) Six genes were selected to construct the prognostic model by the Boruta algorithm. (**E**) Thirteen genes were selected to construct the prognostic model by the SVM-RFE algorithm. (**F**) Intersection feature selection between LASSO, SVM-RFE algorithm, and Boruta algorithm and their individual components.

TCGA transcriptomics were performed in 143 GBM patients using the WGCNA method to acquire gene modules related to NCPS. The soft threshold was set to 12, the minimum number of modules was set to 80, deepSplit was set to 2, and the modules with similarity lower than 0.25 were merged to derive a total of 15 non-grey modules (Figure 3A). In non-grey modules, a strong association was found between Meblue, Mebrown, and Meyellow and NCPS (Figure 3B). Genes from the three modules were selected for subsequent analysis.

### 3.3. Selection of Prognostic Candidate Genes by Machine Learning Algorithms

To begin with, differential genes between high and low expression populations were acquired according to the NCPS gene scores from the previous single-cell dataset. It was then intersected with the NCPS-related genes obtained in the WGCNA to yield a total of 1437 genes. By matching the TCGA with the crossover genes obtained in the previous step, eventually, all genes analyzed in the previous step were allowed for subsequent calculations. In the following analysis, we take the TCGA cohort as our training set, univariate Cox analysis was set at *p* < 0.01, and 18 genes associated with patient prognosis were initially obtained. Then, the random seed was set to 2022 and the results of the regression revealed that the contraction of the lasso stabilized when the number of variables was 14, with the smallest deviation in the score and the best LAMDA of 0.062 (Figure 3C). The Boruta algorithm set the same random seeds and outcome variables as the previous two algorithms, and obtained three genes that the algorithm considered as definitively correlated with survival time, called “Confirmed”; three genes that were considered potentially correlated, described as “Tentative”; the rest of the variables were not considered relevant. In order to include more variables, “Confirmed” and “Tentative” were selected for inclusion in the final results and plotted for display (Figure 3D). SVM-RFE used survival time as the outcome variable, and the results of the 10-fold validation showed that minimal root mean square error (RMSE) was obtained when the number of genes was 13, outputting the above genes as the result of SVM-RFE (Figure 3E). The results of the above algorithms were presented in Venn diagrams and the overlapping results were selected as the final candidate features for inclusion in the construction of the model so that we obtained four genes (Figure 3F). They are CTSD, AP1S1, YWHAG, and IER3, respectively. The respective optimal results of these three algorithms and the intersecting genes are summarized in Table S3.

### 3.4. Construction and Validation of NCPS-Related Prognostic Model

**Figure 4:**
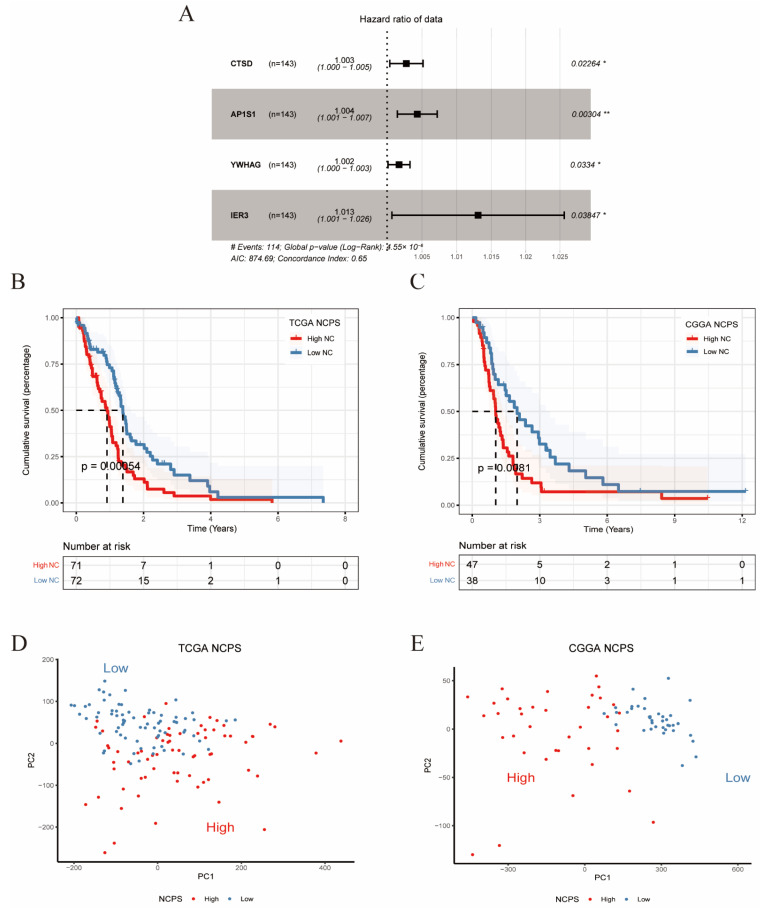
Multivariate Cox and survival analysis. (**A**) Hazard ratio and *p*-value of constituents involved in multivariate Cox regression and some parameters of the signature. (**B**) Survival analysis of TCGA cohort. The prognosis was significantly worse in the high-NCPS group (*p* < 0.001). (**C**) Survival analysis of CGGA cohort. The prognosis was significantly worse in the high-NCPS group (*p* < 0.01). (**D**,**E**) PCA analysis in TCGA cohort and CGGA cohort. It was found that the model could group GBM patients well in both the training cohort and the validation cohort. * *p* < 0.05; ** *p* < 0.01.

Based on the results of multivariate Cox regression analysis, the four genes previously analyzed were still chosen to construct prognostic signatures (Figure 4A). For this signature, the concordance index = 0.65, Akaike information criterion (AIC) = 874.69, and *p*-value = 4.55 × 10^−6^. The following equation was applied to construct the risk score formula: NCPS = (0.002758577 * Expr CTSD) + (0.00431492 * Expr AP1S1) + (0.001704931 * Expr YWHAG) + (0.013004668* Expr IER3). Where each coefficient is derived from the results of multivariate Cox regression. All four genes in the formula were considered risk factors for HR > 1 (Figure 4A). A score was assigned to the prognosis of each GBM patient based on the above formula, and all patients were divided into two groups, named the high-risk or low-risk group, according to the median of this score. In the figure, survival time was more beneficial for patients with low-risk scores (*p* < 0.001). Surprisingly, in the validation set (CGGA), we observed similar results, namely a significantly worse outcome in patients with high NCPS (*p* < 0.01, Figure 4B,C). Furthermore, we analyzed the ROC curves of the training and validation cohorts (Figure S1). Finally, PCA analysis was used to analyze the high-risk and low-risk of the training and validation groups, which indicated that the model could better group the GBM patients. (Figure 4D,E).

### 3.5. Immune Infiltration and Mutation Landscape

**Figure 5:**
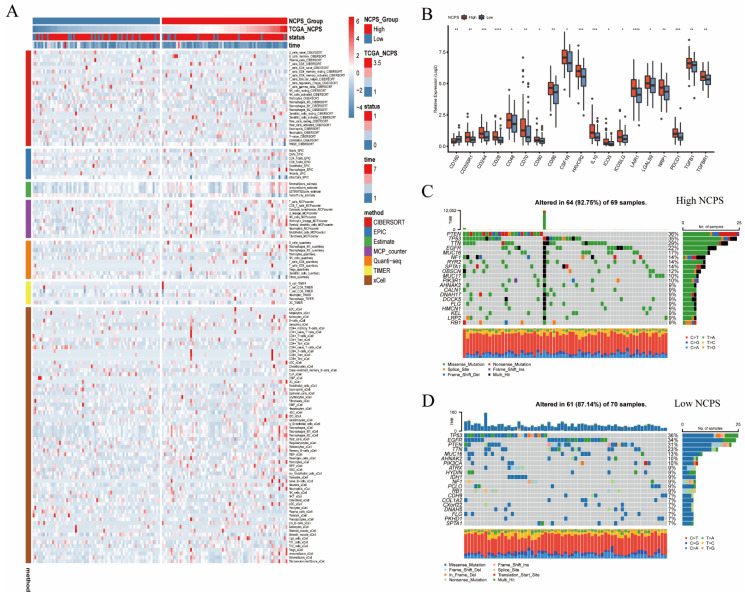
Immune infiltration analysis and mutation landscape. (**A**) Heat map of immune cell infiltration in the high NCPS group and low NCPS group. (**B**) Expression of immune checkpoint-related genes in the high NCPS group and low-NCPS group. The results showed that the expression trend of immune checkpoint-related genes was higher in the high NCPS group. (**C**) Mutation landscape of TCGA cohort in the high-NCPS group. (**D**) Mutation landscape of TCGA cohort in the low-NCPS group. * *p* < 0.05; ** *p* < 0.01; *** *p* < 0.001; **** *p* < 0.0001.

The above findings demonstrated that there was a significant difference in NCPS survival between different groups of GBM patients. To explore the degree of immune infiltration and its causes between the high-risk and low-risk groups, and to provide a basis for clinical application, we performed immune infiltration and mutation analysis (Figure 5A). Patients in the high NCPS group had a large infiltration of macrophages, with M1 and M2 types predominating. Next, the results of the analysis of genes related to immune checkpoints showed that genes such as LAIR1 and CD28 showed high expression in patients with high NCPS (Figure 5B). It can then be conjectured that patients with high NCPS have a higher degree of immune infiltration, and then patients in the high NCPS group are more likely to benefit from inhibitors of immune checkpoints due to the low response status caused by high immune checkpoint genes. Then, we analyzed the top 20 mutated genes between the different groups (Figure 5C,D). It was found that the percentage of top 20 mutations was 92.75% in high NCPS and 87.14% in low NCPS. The main mutation type at both groups of base sites was a mutation from cytosine to thymine. In patients with high NCPS, the PTEN gene was most prominently mutated, while the TP53 gene was the most notably mutated in patients with low NCPS.

### 3.6. Cell Localization of Four Modeling Genes

**Figure 6:**
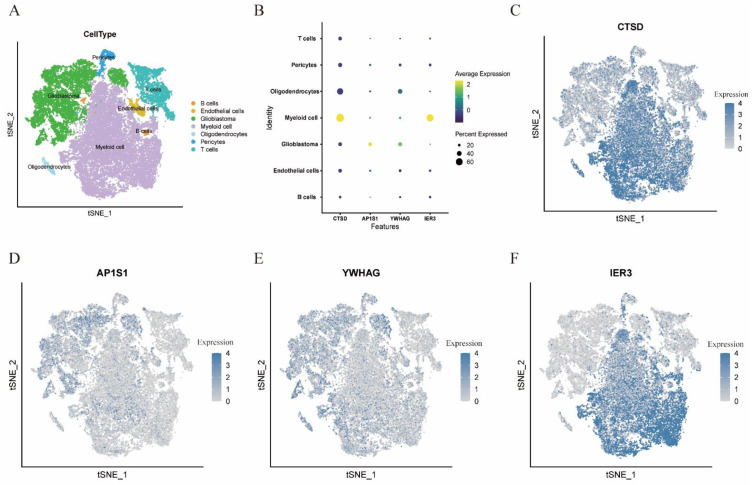
Single-cell sequencing analysis to explore the cell localization of 4 modeling genes. (**A**) The location of different cell types in GBM. (**B**–**F**) CTSD expression was highest in myeloid cells, high expression of AP1S1 was shown in glioblastoma cells, YWHAG was mainly expressed in glioblastoma cells, oligodendrocytes, and myeloid cells, and IER3 was predominantly expressed in myeloid cells.

After constructing the model, we tried to obtain more insight into the expression of the screened NCPS-related genes in different cell types, which we explored in single-cell sequencing analysis. As shown in Figure 6A–F, CTSD expression was highest in myeloid cells, high expression of AP1S1 was shown in glioblastoma cells, and YWHAG was mainly expressed in glioblastoma cells, oligodendrocytes, and myeloid cells, and IER3 was predominantly expressed in myeloid cells.

### 3.7. The Construction of a Nomogram

**Figure 7:**
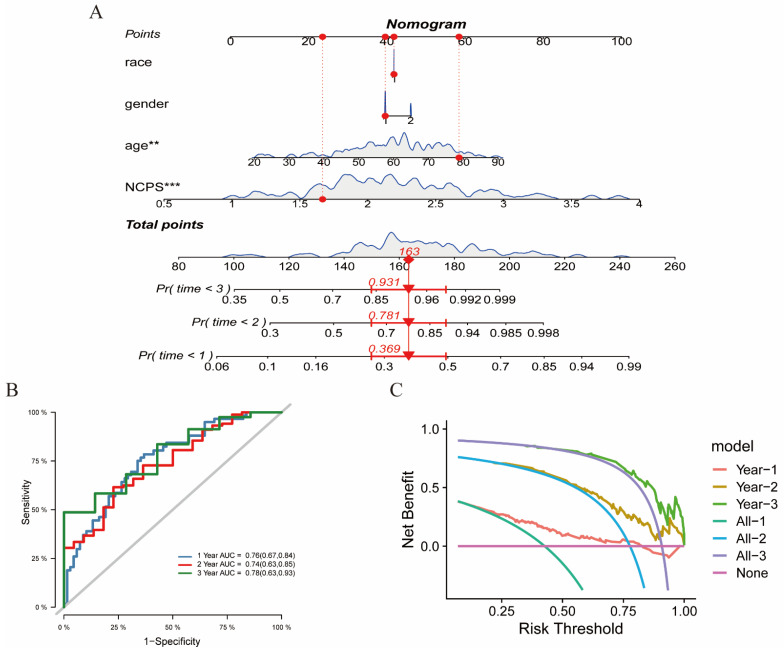
The construction of a nomogram. (**A**) Nomogram of patient “TCGA-02-0047”. The mortality rates at 1, 2, and 3 years were estimated from the “TCGA-02-0047” patients by gender, age, race, and NCPS scores of 0.369, 0.781, and 0.931, respectively. (**B**) ROC curve of the nomogram. The area under the curve (AUC) in 1, 2, and 3 years were 0.76, 0.74, and 0.78, respectively. (**C**) Decision curve analysis. This method is effective in predicting the prognostic survival time of patients. ** *p* < 0.01; *** *p* < 0.001.

By analyzing the clinical data and NCPS values, a nomogram was constructed to better evaluate the risk of GBM patients (Figure 7A). The mortality rates at 1, 2, and 3 years were estimated from the “TCGA-02-0047” patients by gender, age, race, and NCPS scores of 0.369, 0.781, and 0.931, respectively. The nomogram can better evaluate the patient’s risk and guide future clinical decisions. This method was analyzed using the ROC method to evaluate its model accuracy (Figure 7B). The AUC values were found to be 0.74, 0.76, and 0.78 for 1, 2, and 3 years. In addition, a decision curve analysis was also performed based on the area of 1, 2, and 3 years and the horizontal axis of None. The results indicate that this method is effective in predicting the prognostic survival time of patients and has some reference value for clinical treatment decisions (Figure 7C).

## 4. Discussion

Despite partial clinical advances, GBM remains the most aggressive and common malignancy of the brain [35]. Due to heterogeneity, rapid appreciation, and aggressiveness, patients often have a low average survival of 12–15 months [36]. An increasing number of patients with advanced solid tumors have seen some improvement in prognosis, stemming from the translation of immunotherapy to clinical oncology. GBM is unlike other extracranial solid tumors, as peripheral conventional immune cells seem to be less appreciated intracranially and the brain is mostly in a state of immune quiescence; on the other hand, the brain becomes very poorly tolerated when an intracranial lesion arises, especially when an immune attack from a tumor-like GBM occurs, making it fascinating to explore the immune microenvironment of GBM [37]. In the current study, we constructed a prognostic signature associated with NCPS based on machine learning algorithms through extensive analysis of GBM in single-cell sequencing data and TCGA transcriptome data. Four genes were finally screened as having prognostic values, namely CTSD, AP1S1, YWHAG, and IER3. We then performed a risk score on their constructed models to classify GBM patients into high- and low-risk groups. The results of the survival curves explicitly showed that patients with NCPS-related high risk scores had a worse prognosis (*p* < 0.001).

NCPS plays a critical role in the regulation of cancer biology, including tumorigenesis and cancer metastasis [38]. Therapeutic strategies targeting apoptosis resistance in tumor cells have led to the increasing research status of NCPS in tumors. The specific process of NCPS involves activation of RIP1 by TNF-α/TNFR1 when caspase-8 is cleared or inhibited by mutation or infection and the subsequent recruitment of RIP3 through the RIP homotypic interaction motif (RHIM) [39]. The interaction of these two proteins results in the phosphorylation of RIP3, which activates its kinase in proper sequence, resulting in increased phosphorylation of RIP3 and downstream MLKL. The combinatorial interaction of RIP1–RIP3 and RIP3–MLKL collectively forms a necrosome complex to execute NCPS [40,41]. Several studies have shown that the effect of NCPS on tumors appears to be a double-edged sword: on the one hand, key molecules associated with NCPS promote cancer progression and reduce patient survival [42,43], and on the other hand, several studies have demonstrated that promoting NCPS can effectively inhibit tumor growth [44,45,46]. Although studies on the effects of NCPS on CNS tumor processes are limited, the available information suggests a possible link between genes related to the necroptotic pathway and the clinical behavior of GBM. For example, a significant reduction in overall survival (OS) in GBM under P53-induced inhibition is inextricably linked to the high expression of RIPK1 [47]. Nevertheless, the effect of NCPS on GBM has not been fully elucidated. To date, studies related to NCPS in GBM have been relatively scarce. In the current study, our results showed that four NCPS-related genes have predictable value in the prognosis of the GBM risk. The survival curves explicitly showed that patients with NCPS-related high risk scores had a worse prognosis. This suggests that NCPS may play a pro-cancer role in GBM, which is consistent with the results of other analyses [47,48]. Our study provided a basis for the prediction of NCPS-related genes in GBM and lays a foundation for further study of their prognosis.

The tumor microenvironment plays a pivotal role in tumors. In addition to the process of tumor development, it also has a remarkable impact on immunotherapy outcomes and overall patient survival [49]. In addition to the influence of the tumor and its surrounding microenvironment, the systemic immune-inflammatory status has also received increased attention [50,51]. Studies have found that elevated neutrophils, lymphocytes, and platelets in the blood of patients with tumors are associated with aggressiveness and poor prognosis of solid tumors [52,53]. In studies related to GBM, systemic immune-inflammatory index, which uses blood cells as a marker for detection, has been proven to be significantly associated with patient prognosis [54]. Interestingly, this index can also assess the impact of different types of surgery on the prognosis of GBM patients [55]. The complicated immune environment, involving immune cell distribution within and around the tumor, immune cell composition, and the overall immune environment of the GBM, can all have an impact on the effectiveness of immunotherapy and even the malignancy of the tumor [56]. We have researched the immune microenvironment of GBM and found that NCPS may be associated with macrophage infiltration in GBM, particularly in the M1 and M2 subtypes. Previous studies have also demonstrated the close association of macrophages not only with glioma survival and prognosis but also with their role in apoptosis [57]. Furthermore, in our follow-up study, we found that patients with highly expressed genes related to immune detection sites are more sensitive to this immunotherapy. This indicates that the model we made not only could determine the survival prognosis of patients but also could reflect the immune infiltration of the tumor. In addition, the comparison results between different risk groups showed that the number of mutated genes in the high-risk group was significantly higher than that in the low-risk group. This may be due to the deterioration of the microenvironment due to the mutation of immune-related genes, which, in turn, promotes tumorigenesis. Therefore, our results may provide new evidence for the immunotherapy of GBM.

The CTSD gene, a member of the peptidase A1 family, has been shown to be highly expressed in the breast cancer tumor microenvironment and is strongly associated with the survival of breast cancer patients [58]. Recent experiments have identified a role for CTSD in GBM: upregulation of CTSD expression contributes to enhanced radio-resistance of GBM cells, while inhibition of CTSD significantly reduces the migration ability of GBM cells [59]. As a member of the bridging protein (AP) family that coordinates various transports in the intracellular membrane pathway, AP1S1 and its related genes were shown to be associated with immune response, lysosomal vesicle transport, and protein presentation [60]. In previous bioinformatics analyses, investigators found that AP1S1 was highly expressed in GBM and associated with survival prognosis [61]. Our findings, which identified AP1S1 as an independent prognostic indicator in GBM, are consistent with previous studies. YWHAG, one of the 14-3-3 family isoforms, is involved as a regulatory molecule in a variety of cellular processes, including cell survival and apoptosis, protein transport, and cell cycle regulation [62,63]. Researchers have found that YWHAG was significantly overexpressed in GBM, head, and neck squamous cell carcinoma, and breast and gastric cancer [64,65,66]. IER3 belongs to a stress-inducible gene, also known as IEX-1, which is classified as a family of immediate early response genes. IER3 plays a complex, and to some extent contradictory, role in cell cycle control and apoptosis [67]. Current studies on IER3 in tumors have also confirmed that it is expressed in opposite ways in different tumors [68,69], so the exact mechanism and function of this gene remains to be explored. In this study, the results showed that CTSD and IER3 were predominantly expressed in myeloid cells, high expression of AP1S1 was shown in glioblastoma cells, and YWHAG was mainly expressed in glioblastoma cells, oligodendrocytes, and myeloid cells. This corroborates our previous evidence of association of NCPS with macrophages in immune infiltration. The value shown by these four genes in GBM patients deserves deeper investigation.

The previously published GSE182109 dataset explored the heterogeneity and immune infiltration of gliomas, including low-grade gliomas, and primary and recurrent glioblastomas, by single-cell sequencing analysis. The investigators analyzed the GBM immune microenvironment and classification of functional immune cell subtypes, on the basis of which specific immune checkpoints were inspected and screened, and S100A4 was identified as a potential target for immunotherapy [31]. In this study, primary glioblastoma data from the GSE182109 dataset were first subjected to further single-cell analysis, which divided them into two groups with different NCPS states. This is a useful reference for studying the heterogeneity of NCPS states in GBM. Afterward, a prognostic model was constructed based on differentially expressed genes in the cell population. This model was tested using TCGA survival data. One of the strengths of this model is its ability to accurately assess the prognosis of GBM patients. This study is the first to apply single-cell clustering analysis to predict the prognosis of GBM cell necroptosis, which may not only provide a new perspective to study its regulated cell death different from transcriptome analysis, but also provide a new angle for clinical treatment. Admittedly, there are certain shortcomings in the present study. Due to the limitations of current technology, GBM patients are still under-explored at the single-cell level on various immune cell subtypes with NCPS. In addition, there is no standardized assay for clinical screening of the expression of the NCPS-related genes we obtained, and we know too little about the underlying mechanisms of the complex functions of NCPS, which we hope will be investigated more in the future scientific work.

## 5. Conclusions

In summary, we developed a predictive model for glioblastoma NCPS genes. These genes may be closely related to the mechanism of NCPS in GBM. The expression of these four genes in specific cells, such as myeloid cells and glioblastoma cells, makes us more curious about the specific function of NCPS on immune cells and tumor cells. These results contribute to further understanding of the molecular mechanisms of GBM regulation. The construction of this model allows us to evaluate GBM from a new perspective, in addition to the tumor microenvironment of GBM, and to design effective combination therapies based on cell type-specific expression.

## Data Availability

Data are contained within the article.

## Supplementary Materials

The following supporting information can be downloaded at: https://www.mdpi.com/article/10.3390/brainsci12080988/s1, Figure S1: ROC curves of the training and validation cohorts; Table S1: Baseline of GBM patients; Table S2: Cell Types Annotation; Table S3: Machine learning algorithms and results.
