# Supplementary figures and images for: A Novel Necroptosis-Related Prognostic Signature of Glioblastoma Based on Transcriptomics Analysis and Single Cell Sequencing Analysis
Source: Brain Sci. 2022 Jul 26;12(8):988. doi: 10.3390/brainsci12080988 (PMC9460316; doi:10.3390/brainsci12080988)

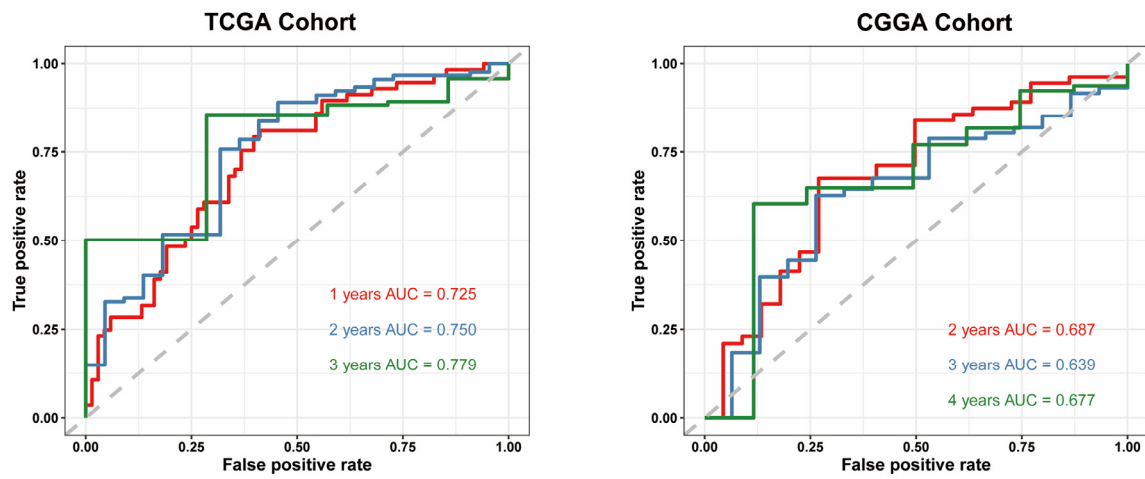

**Figure S1.** ROC curves of the training and validation cohorts.

Supplement: Supplementary file 1 [file brainsci-12-00988-s001.zip › Figure S1.pdf]
